# Effects of Perturbations to Balance on Neuromechanics of Fast Changes in Direction during Locomotion

**DOI:** 10.1371/journal.pone.0059029

**Published:** 2013-03-18

**Authors:** Anderson Souza Oliveira, Priscila Brito Silva, Morten Enemark Lund, Leonardo Gizzi, Dario Farina, Uwe Gustav Kersting

**Affiliations:** 1 Center for Sensory-Motor Interaction, Department of Health Science and Technology, Aalborg University, Aalborg, Denmark; 2 The CAPES Foundation, Brazilian Education Ministry, Brasilia, Brazil; 3 Department of Mechanical and Manufacturing Engineering, Aalborg University, Aalborg, Denmark; 4 Pain Clinic Center for Anesthesiology, Emergency and Intensive Care Medicine University Hospital Göttingen, Göttingen, Germany; 5 Department of Neurorehabilitation Engineering, Bernstein Focus Neurotechnology Göttingen, Bernstein Center for Computational Neuroscience, University Medical Center Göttingen, Georg-August University, Göttingen, Germany; Universidad Europea de Madrid, Spain

## Abstract

This study investigated whether the modular control of changes in direction while running is influenced by perturbations to balance. Twenty-two healthy men performed 90° side-step unperturbed cutting manoeuvres while running (UPT) as well as manoeuvres perturbed at initial contact (PTB, 10 cm translation of a moveable force platform). Surface EMG activity from 16 muscles of the supporting limb and trunk, kinematics, and ground reaction forces were recorded. Motor modules composed by muscle weightings and their respective activation signals were extracted from the EMG signals by non-negative matrix factorization. Knee joint moments, co-contraction ratios and co-contraction indexes (hamstrings/quadriceps) and motor modules were compared between UPT and PTB. Five motor modules were enough to reconstruct UPT and PTB EMG activity (variance accounted for UPT  = 92±5%, PTB = 90±6%). Moreover, higher similarities between muscle weightings from UPT and PTB (similarity = 0.83±0.08) were observed in comparison to the similarities between the activation signals that drive the temporal properties of the motor modules (similarity = 0.71±0.18). In addition, the reconstruction of PTB EMG from fixed muscle weightings from UPT resulted in higher reconstruction quality (82±6%) when compared to reconstruction of PTB EMG from fixed activation signals from UPT (59±11%). Perturbations at initial contact reduced knee abduction moments (7%), as well as co-contraction ratio (11%) and co-contraction index (12%) shortly after the perturbation onset. These changes in co-contraction ratio and co-contraction index were caused by a reduced activation of hamstrings that was also verified in the activation signals of the specific motor module related to initial contact. Our results suggested that perturbations to balance influence modular control of cutting manoeuvres, especially the temporal properties of muscle recruitment, due to altered afferent inputs to the motor patterns. Furthermore, reduced knee stability during perturbed events may be related to overall control of lower limb muscles.

## Introduction

Stability during human locomotion is continuously challenged, requiring mechanisms that integrate visual, vestibular and somatosensory inputs [1]–[3]. In sport activities, such as running and sudden changes in directions (i.e., cutting manoeuvres), the attention is not essentially focused on balance control since there are other relevant aspects concerning motor performance and there may be unpredictable changes in the environment (i.e., the interaction between the practitioner and the opponents/surfaces). It is known that there are direct connections between hamstring muscles and the anterior cruciate ligament [4] and that hamstrings muscles contribute to knee joint stability while running/cutting [5], [6]. Nonetheless, changes in the environment are risk factors for lower limb injuries [7]–[10] since changes in the muscular activation may reduce the above mentioned protective mechanisms [5], [6], [11]–[14]. Perturbations such as slips usually occur shortly after initial contact of cutting manoeuvres, and may expose the lower limb joints to injury risks [15]. Knowing the central nervous system (CNS) strategies elicited during perturbations to balance, based on experimental data directly related to the motor gesture, may be relevant to advance injury prevention.

Muscular coordination for changes in direction during running has been considered a crucial factor for injury prevention [16], [17]. In addition to the study of the activity of each muscle, muscle coordination can also be investigated by a reduction of dimensionality, focusing on less primitive signals than the active muscles [18]. Motor modules (also called muscle synergies) are defined as sets of muscles recruited in specific time-varying profiles [18]–[20]. With this approach, recently, Oliveira and co-workers described the modular organization of neural inputs to the muscles during changes in direction [21]. Perturbations to balance while walking elicit specific and rapid neural strategies to avoid falls [22], [23], but the modulation of walking is predominantly preserved [20], [24]. On the other hand, the activation signals which dictate the timing for the recruitment of motor modules are substantially altered by perturbations [20], suggesting that the afferent input must play essential role during perturbations.

Therefore, the reduced protection caused by altered muscular activation during changes in direction might be linked to changes in the afferent participation on the task performance. In the present investigation we aimed to verify whether perturbations delivered at initial contact could influence the modular control and stability of the lower limb joints. Perturbations were elicited in the original direction of the running prior to the turning, so that the most probable foot trajectory during perturbation could be imitated. We hypothesized that motor modules extracted during changes in direction could be influenced by small perturbations to balance, especially by the altered afferent components evoked by perturbations. Motion analysis and factorization analysis were used in order to understand the perturbation effects and suggest neural mechanisms related to the described postural responses.

## Methods

### 2.1. Subjects

Twenty-two healthy men (age: 28±4 yrs; body mass: 71±10 kg; body height: 171±7 cm) volunteered for the experiment. All subjects were recreational practitioners of team sports (soccer, basketball, handball, ice hockey). They had no known history of neurological or motor disorder. All subjects provided written informed consent before participation and the procedures were approved by the ethical committee of Northern Jutland (N-20100042).

### 2.2. Experimental Setup

**Figure 1:**
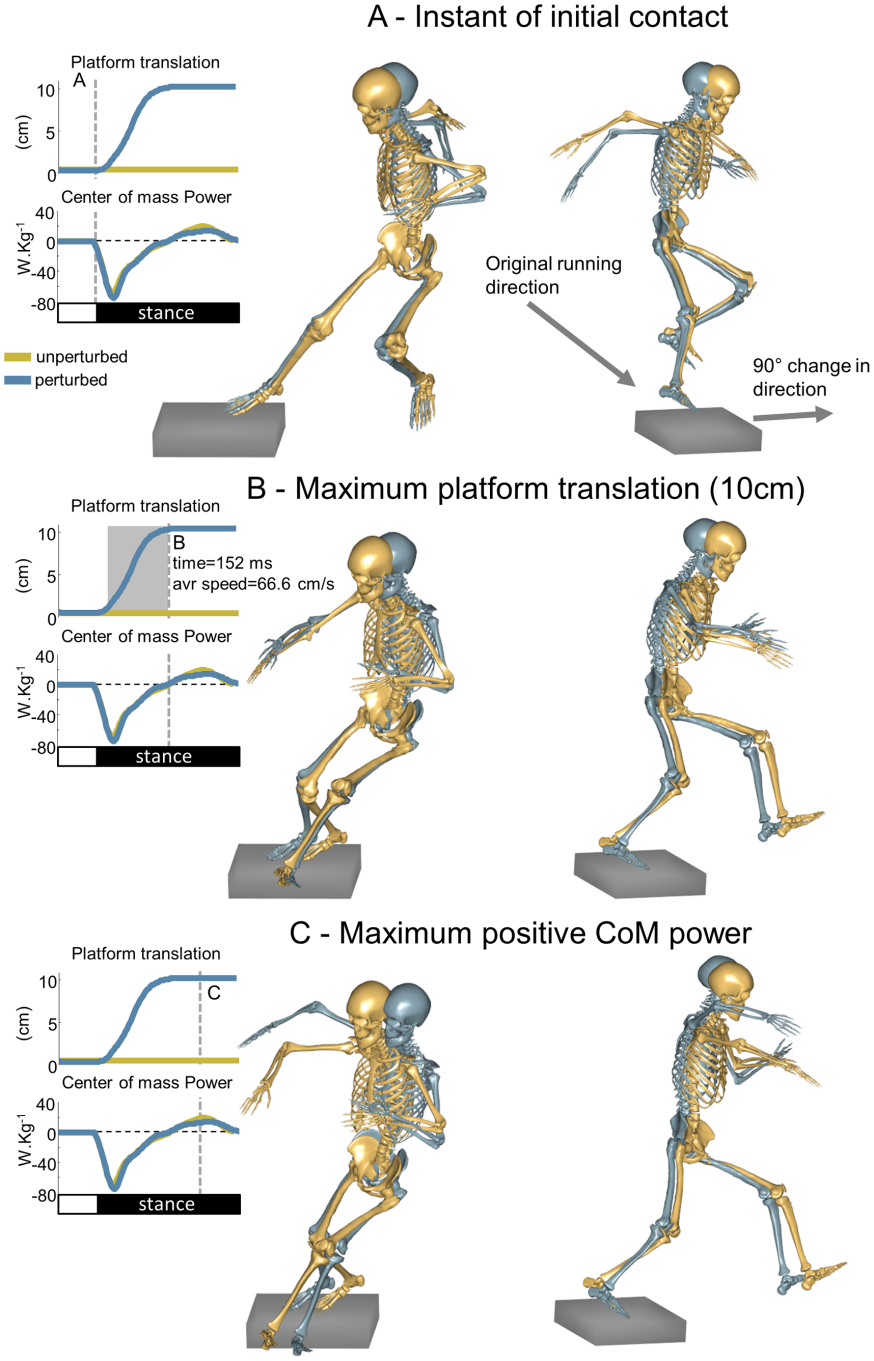
Three dimensional models from a representative subject performing unperturbed (*yellow*) and perturbed (*blue*) cutting manoeuvres. The comparison between the two conditions was emphasized in three events. A) The time of initial contact, note that there is no difference in the lower limbs kinematics, indicating consistent inter-trial behavior. B) The time 152 ms (*grey area in the translation plot*) after initial contact. This time corresponds to 10 cm translation of the platform in the perturbed trial. Note the perturbed model shows the right foot forwarded in relation to the unperturbed model, as well as a more abducted hip position. C) The time of maximum power output. The maximum power generated during the stance phase is slightly lower for the perturbed condition. At this instant, the trunk position is influenced by the perturbations as well as the contralateral leg.

Subjects were asked to perform repeated running trials with a 90° change in direction (cutting manoeuvres) during a single session. The task consisted in running from 6–7 meters away of a moveable force platform, aiming to step with the right foot onto the plate, turn 90° to the left and continue running (see Figure 1 for illustration). Initially, 10–15 familiarization trials were required and instructions to accelerate in a straight path towards the force platform and turn as fast as possible to the left were provided. Subsequently, 11 cutting manoeuvres were recorded with 40–60 s rest intervals within each trial to reduce the effects of fatigue. Without any previous warning, there was a perturbation elicited at the initial contact to the moveable force platform during the 11^th^ trial. The perturbation consisted of a 10-cm translation lasting 150 ms (average speed 66.6 cm/s) in the original running direction. Subjects wore the same type of court shoes (FZ 2600W, FORZA®, Brønderslev, DK) in order to reduce the effects of different footwear on the measurements.

### 2.3. Data collection

#### Kinematics

Retroreflective spherical markers were placed bilaterally each side of the subject to the skin overlying the following landmarks bilaterally: calcaneus, first and fifth metatarso-phalangeal joint, lateral malleolus, lateral condyle; greater trochanter, anterior superior iliac spine, posterior superior iliac spine and acromion. In addition, one marker was placed on the seventh cervical vertebrae, upper and lower endpoint of sternum (suprasternal notch and xiphoid process). Extra markers were placed bilaterally on lower extremity segments: one on the thigh, four on the shank and one on the upper arm, serving as tracking markers to define the 3D motion. Marker positions were tracked with a motion analysis system with eight infrared digital video cameras (Oqus 300 series, Qualisys, Gothenburg, Sweden). The kinematic data were recorded with a sampling frequency of 256 Hz and synchronized with the EMG and kinetic recordings. Subjects wore full stretch pants covering the EMG cables to avoid movement artifacts.

#### Kinetics

The vertical (*Fz*), anterior-posterior (*Fy*) and medial-lateral (*Fx*) ground reaction forces and the corresponding reaction moments (Mx, My, Mz) were recorded at 1024 Hz by a force platform (AMTI, OR6-5, Watertown, MA) constructed over a hydraulic system [25]. Software developed on the Labview platform (MrKick II, Aalborg University, Aalborg, Denmark) was used for data recording. Using a feedback electric circuit, the *Fz* force also served as trigger to initiate the force plate movement.

#### Electromyography

Surface EMG signals were recorded in bipolar derivations with pairs of Ag/AgCl electrodes (Ambu Neuroline 720 01-K/12; Ambu, Ballerup, Denmark) with 22 mm of center-to-center spacing. Prior to electrode placement the skin was shaved and lightly abraded. The EMG signals were amplified with a gain of 2,000 (EMG-USB, LISiN; OT Bioelettronica, Turin, Italy), sampled at 2,048 Hz (12 bits per sample), band-pass filtered (second-order, zero lag Butterworth, bandwidth 10–500 Hz). A reference electrode was placed on the right wrist. The EMG signals were recorded from the following muscles of the right side according to the SENIAM recommendations [26] and previous literature [20], [21], [27]: tibialis anterior (TA), peroneus longus (PER), soleus (SOL), gastrocnemius medialis (GM), vastus medialis (VM), vastus lateralis (VL), rectus femoris (RF), long head of biceps femoris (BF), semitendinosus (ST), adductor muscles (ADD), gluteus medius (GME), gluteus maximus (GMA), tensor fascia latae (TFL), erector spinae at L1 (ESP), rectus abdominis (RAB) and external oblique (EOB).

### 2.4. Data Analysis

The body of the subjects was modeled as an interconnected chain of rigid body segments: foot, shank, thigh, pelvis, trunk and arms as described by Andersen and co-workers [28]. Joint angles are calculated as the three rotations of the distal segment with respect to the proximal. The first rotation, flexion (ankle: dorsi flexion), is around the medial/lateral axis of the proximal segment, the last rotation, internal rotation, is around the inferior/superior axis in the distal segment, and adduction (ankle:inversion) is the intermediate rotation around the floating axis perpendicular to the first and the last axis. All angles are calculated under the assumption that all segments (thigh, shank, and foot) are rigid bodies. Segment coordinate systems and the procedure for calculating joint angles follow the specification of the International Society of Biomechanics (ISB) [29] Joint moments are the external joint moment vector projected onto the three rotation axis.

The trunk center of mass, joint angles and angular velocities between segments were analyzed in the AnyBody Modeling System 5.1 (Anybody Technology, Aalborg, Denmark). The left initial contact was defined from the foot kinematic data, whereas the end of the stance phase for the right leg was determined by the force plate recordings (when the vertical ground reaction force exceeded 20 N). The body center of mass (CoM), CoM power (CoMp: sum of ground reaction forces times CoM velocity [watts/body weight]), joint angles and joint moments were calculated during the period of contact to the force platform.

#### Signal processing

**Figure 2:**
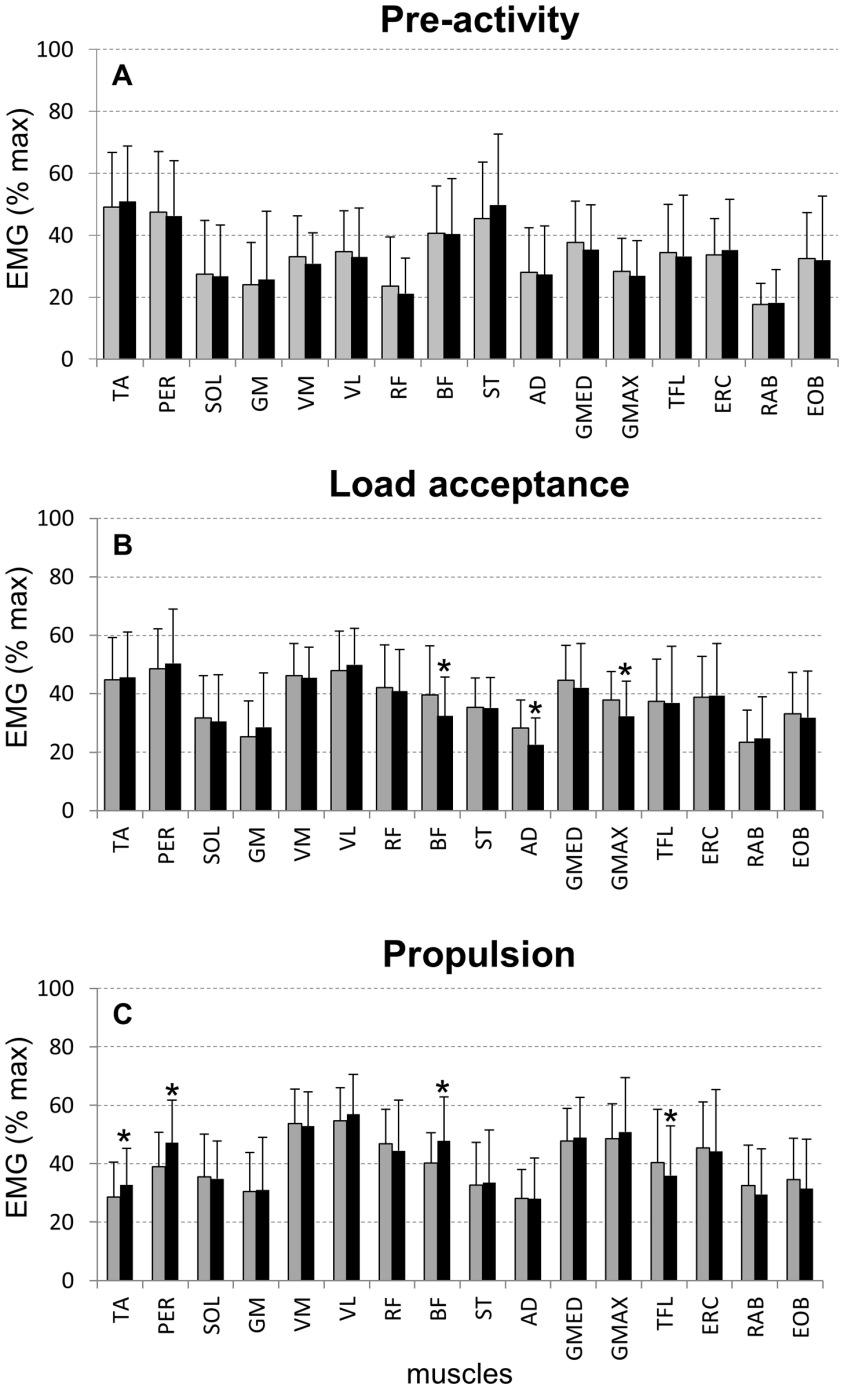
Mean (SD) EMG amplitude 10 ms before initial contact (A), during load acceptance phase (B) and propulsion phase (C) of unperturbed (*grey*) and perturbed cutting manoeuvres (*black*). * denotes significant difference in relation to the perturbed condition (p<0.05).

The segmentation for EMG factorization was defined from the left initial contact prior the right foot step on the force platform to the end of the stance phase on the force platform. Prior to any analysis, EMG signals were band-pass filtered (20–500 Hz). After segmentation, the surface EMG signals from the 16 muscles were full-wave rectified, low-pass filtered (10 Hz) and time-normalized in order to obtain 200 data points for one gait cycle [19], [30]. For each individual, EMG activity for each muscle was normalized with respect to the peak activity found among all trials (unperturbed and perturbed cutting manoeuvres), therefore varying from 0 to 1. For each subject, non-negative matrix factorization (NMF) [19], [31] was applied for each trial in order to identify motor modules and activation signals (Figure 2).

#### Motor module model

The EMG signals recorded from *M* muscles were indicated as:
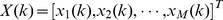
(1)where *x_M_(k)* is the activity of the *m*th muscle at the time instant *k*. The activation signals *P(k)* were indicated as (*N<M*):
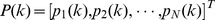
(2)


The relation between *X(k)* and *P(k)* is described as follows:
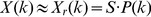
(3)where *X_r_(k)* is the muscle activity vector reconstructed by the factorization. In Eq. (3), the EMG *X(k)* are obtained by linear transformation of the activation signals *P(k)* with gain factors *s_mn_*. The matrix whose columns were the weights of each activation signal (expressed as an arbitrary unit) for each muscle is denoted as *S* in Eq. (3) and will be referred to as the motor module matrix [32].

#### Dimensionality

The number of motor modules *N* needed for accurate description of the movement was assessed by the dimensionality analysis proposed by [19] separately for the perturbed (PTB) and unperturbed cutting manoeuvres (UPT). According to this procedure, the quality of reconstruction of the muscle activation pattern is analyzed as a function of the number of modules and the minimum number of modules is identified as the point in which this curve pronouncedly changes its slope [19]. In addition to this criterion, a minimum threshold for reconstruction quality was set at 80%. For quantifying the quality of reconstruction, the estimated muscular activation pattern was compared with the recorded pattern by means of the variance accounted for (VAF) value, defined as the variability that can be explained by the model: VAF  = 1 – SSE/SST, where SSE (sum of squared errors) is the unexplained variability and SST (total sum of squares) is the pooled variability of the data.

After computation of the reconstruction quality, the motor modules for each subject were extracted from the concatenation of all UPT trials Similarities among the different subjects were investigated for motor modules and activation signals for both UPT and PTB. The motor module matrices were compared by computing the scalar product between pairs of columns, normalized by the product of the norms of each column [19], [33], [34]. Similarities between activation signals were also quantified by scalar products [31], [35]. For both muscle weightings and activation signals the minimum value to consider a comparison “similar” was r>0.80, which is comparable to previous literatures that have used r^2^>0.55 [34]. In addition, the EMG activities from all subjects were concatenated for a given condition, from which motor modules were extracted to represent the whole group of subjects. In this manner, all the variability in the dataset was taken into account. The timing of each activation signal was defined as the time instant of the maximum of the activation signal as a percentage of the cutting cycle. A cross-validation procedure was also used by reconstructing perturbed EMG from fixed muscle weightings or activation signals from unperturbed trials. In this way, it was possible to determine whether activation signals and/or muscle weightings from unperturbed and perturbed conditions were indeed similar. This cross-validation procedure has been adapted from previous investigations in the field [36]–[39]. In addition, we reconstructed EMG signals using two different methods: A) the combination of perturbed muscle weightings with activation signals obtained from randomly generated matrix (i.e., activation signals free to vary) and B) the combination of perturbed activation signals with muscle weightings obtained from randomly generated matrix (i.e., muscle weightings free to vary). The muscle weightings and activation signals free to vary were obtained by iterating 1000 times the NMF update rules [32] only for muscle weightings or activation signals, respectively. Subsequently, the VAF from the comparison between the original perturbed EMG and the two types of reconstructed EMG was calculated.

Kinematic data were low-pass filtered (10 Hz, second-order, zero lag Butterworth) and CoM mass speed (CoM_SPD_) was computed between 200 ms and 100 ms prior to right foot contact to the force platform. Joint angles and joint moments from the hip, knee and ankle were calculated and the peak angles and moments during the load acceptance stance period (defined as period in which the CoM power is negative), and the propulsion period (defined as the period in which the CoM power is positive) were computed for each trial. In addition, the external work (integration of the CoM power) was calculated for the load acceptance (W_LAC_) and propulsion (W_PRP_) period of cutting manoeuvres. Peak horizontal forces in the anterior-posterior direction (HF_AP_) and medial-lateral direction (HF_ML_) were also recorded during the first half of stance period.

Additional EMG analysis was conducted by using the same raw EMG signals from cutting manoeuvres, which was normalized differently in relation to the surface EMG for NMF extraction. For each muscle the maximum EMG during stance period from all PTB and UPT trials was used as index to normalize all trials. Average EMG amplitude (%maximum) was calculated in three time epochs for all 16 muscles: 1) 10 ms before initial contact (PRE); 2) from initial contact until negative peak CoM power during load acceptance (LA); 3) a 50-ms time window around the peak CoM power during the propulsion phase of cutting manoeuvres (PRP). In addition, co-contraction ratio (CCR) and co-contraction index (CCI) for the relationship between knee flexors and extensors were computed for each of the time epochs [40]. The CCR was defined as the average knee flexors EMG activity ((BF+ST)/2) divided by the knee extensors activity ((VM+VL+RF)/3). The CCI was defined as the product of the averaged EMG activation of from all knee flexors and extensors and the CCR.

#### Statistical Analysis

The effects of perturbation on the dependent variables (stance duration, CoM_SPD_, W_LAC_, W_PRP_, HF_AP_, HF_ML_, peak joint angles, peak moments, EMG PRE, LA, PRP, CCI, CCR and peak timing of the activation signals) were investigated using Student's t-test for paired samples. The significance level was set to p<0.05.

## Results

The comparison between PTB and UPT performance of cutting manoeuvres (Figure 1) showed that subjects did not change the approach to perform PTB (Figure 1A), since they had no previous warning on the perturbation event. The platform translation in the direction of the original running increased knee extension and hip and knee abduction (Figure 1B), but no changes in the contralateral limb or trunk position were observed. The most pronounced effects of the perturbation were observed at the instant of peak CoM power generation (Figure 1C), where the trunk position is compromised, causing this subject to raise the arms in order to facilitate balance recovery. In addition, there is a greater knee external rotation in the perturbed knee at this moment.

Perturbations to balance during cutting manoeuvres did not influence stance duration and CoM_SPD_ (p>0.05, Table 1). On the other hand, W_LAC_ and W_PRP_ were reduced for PTB (p<0.05). Greater HF_AP_ were observed in comparison to HF_ML_ for both UPT and PTB (p<0.01). In addition, perturbations to balance induced increases in peak values for both HF_AP_ and HF_ML_ (p<0.05).

**Table 1:** Mean(SD) stance duration (stc_sur), CoM speed 100 ms before initial contact (CoM_SPD_), external work during load acceptance (W_LAC_), propulsion period (W_PRP_) and horizontal forces in the anterior-posterior (HF_AP_) and medial-lateral directions (HF_ML_) for the unperturbed and perturbed cutting manoeuvres.

	Unperturbed	Perturbed
stc_dur (ms)	327.90±5	324.70±5
CoM_speed (m.s^−1^)	2.67±0.4	2.62±0.5
W_LAC_ (W.kg^−1^)	−14.30±3.9	−15.50±5.2*
W_PRP_(W_kg^−1^)	9.90±3.2	7.90±3.3*
HF_AP_ (N.kg^−1^)	10.0±3.0^†^	11.58±3.7^†^*
HF_ML_ (N.kg^−1^)	6.93±2.0	7.30±2.5*

*indicates significant difference in relation to unperturbed cutting (p<0.05); † indicates significant difference in relation to HF_ML_ (p<0.01).

Perturbations to balance did not influence muscle activation immediately before initial contact (p<0.05, Figure 2A). On the other hand, reduced EMG activity during perturbed LA was observed for BF, AD and GMAX (p<0.05, Figure 2B), whereas EMG activity was greater during perturbed PRP for TA, PER, BF and TFL (p<0.05, Figure 2C)

**Figure 3:**
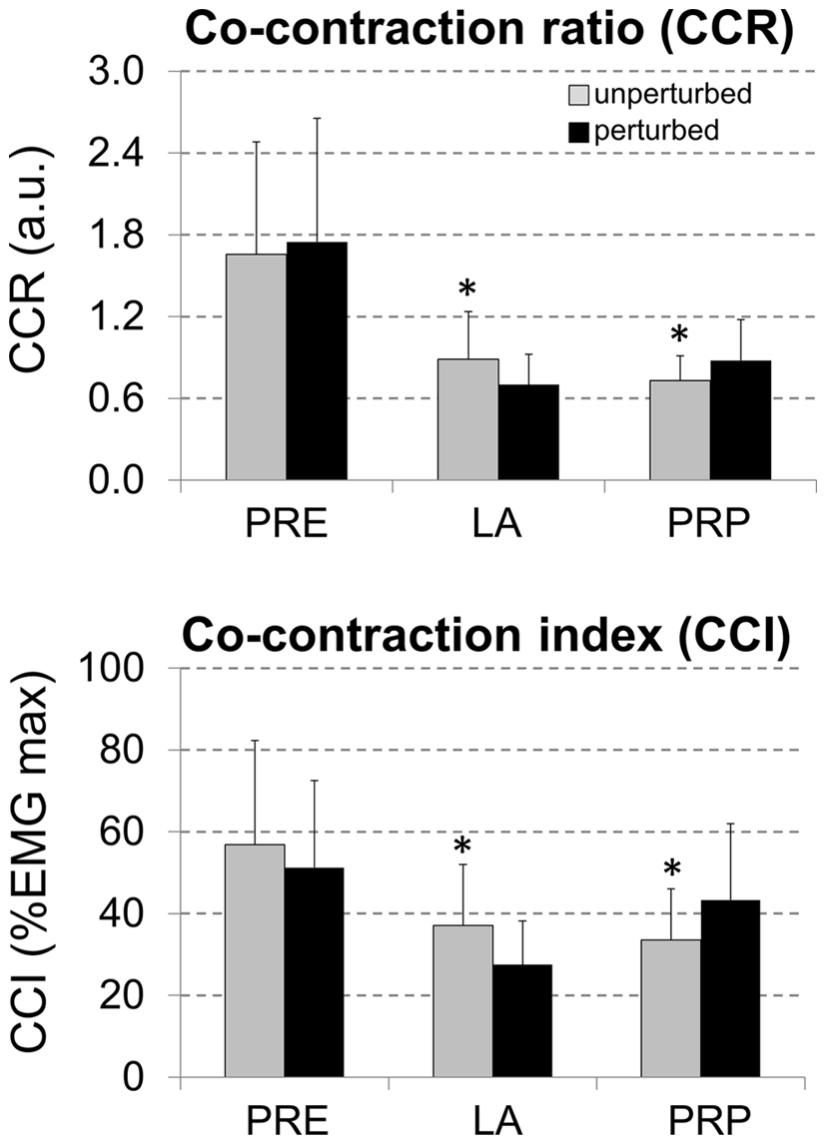
Mean (SD) co-contraction ratios (top) and co-contraction indexed (bottom) 10 ms before initial contact (PRE), during load acceptance phase (LA) and propulsion phase (PRP) of unperturbed (*grey bars*) and perturbed cutting manoeuvres (*black bars*). * denotes significant difference in relation to the perturbed condition (p<0.05).

No effects of perturbation were found for CCI and CCR before initial contact (i-pre10, Figure 3). On the other hand, during i-Abs, both CCI and CCR were reduced when perturbations were elicited (p<0.05). Moreover, both CCI and CCR showed significant increases during i-prop for PTB (p<0.05).

### The effects of perturbation on joint kinematics

**Figure 4:**
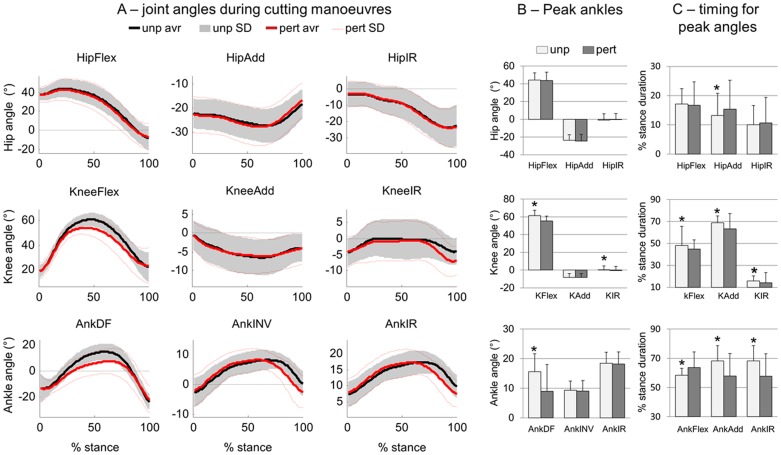
Kinematics of unperturbed (unp) and perturbed (pert) cutting manoeuvres. A) Three dimensional joint angles were extracted from the hip, knee (K) and ankle (Ank). Statistical analysis compared the peak angles (B) and the timing for the peak angles (C). Flex  =  flexion; Add  =  adduction; IR  =  internal rotation; DF  =  dorsiflexion; IN V =  inversion. * denotes significant differences in relation to PERT.

Figure 4A shows the average joint angles for PTB and UPT throughout the stance period. It was observed that joint angles are very similar for the hip joint. On the other hand, knee showed reduced peak flexion (6.1±1.2°), and increased peak external rotation (0.04±3.1° and −0.61±4.0°, respectively, p<0.05, Figure 4B). The ankle joint showed reduced peak dorsiflexion (7.2±1.9°, p<0.05). Towards the end of the perturbed stance period, it was observed that the knee was more externally rotated, and the ankle was more everted and externally rotated in relation to UPT. Concerning the timing of the peaks, there was a verifiable delay in the peak of hip adduction and ankle dorsiflexion due to perturbations (∼4%, Figure 4C, p<0.05). The ankle joint showed earlier peaks for inversion and internal rotation, whereas the knee showed earlier peaks for all directions for PTB. (6–12%, p<0.05).

### The effects of perturbation on joint moments

**Figure 5:**
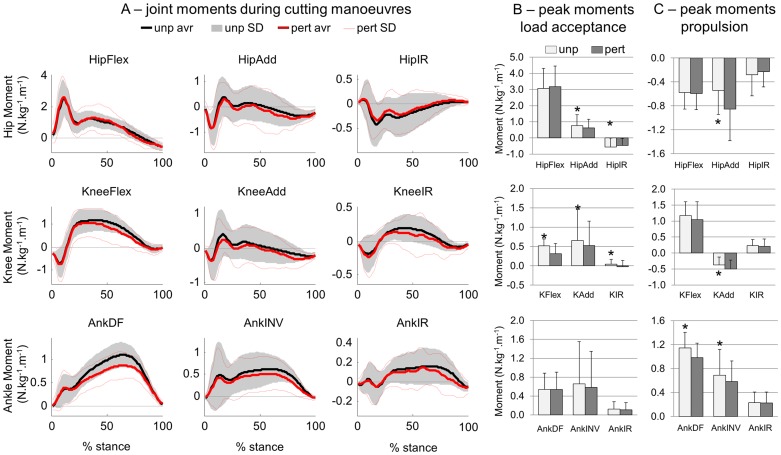
Joint moments of unperturbed (unp) and perturbed (pert) cutting manoeuvres. A) Three dimensional joint moments were extracted from the hip, knee (K) and ankle (Ank). Statistical analysis compared the peak moments during load acceptance period (B) and during propulsion period (C). Flex =  flexion; Add  =  adduction; IR  =  internal rotation; DF  =  dorsiflexion; INV  =  inversion. * denotes significant differences in relation to PERT.

Changes in joint moments were found predominantly in the medial-lateral and flexion-extension directions (Figure 5A). With respect to the load acceptance period (approximately the first 20–25% of the stance period), the peak hip adduction and external rotation moments were reduced (p<0.05, Figure 5B). In the same way, the peak knee flexion, adduction and internal rotation momentswere reduced (p<0.05) during the load acceptance period, with no changes in ankle moments. Increases in hip and knee peak abduction moments during the propulsion period (Figure 5C, p<0.05) were demonstrated. In addition, peak ankle dorsiflexor and invertor moments were reduced during the propulsion period.

### Dimensionality

The analysis of dimensionality from single trials revealed that five motor modules were required to reconstruct unilateral muscular activation for both UPT (average VAF = 92±5%) and PTB (average VAF = 90±6%). On average, VAF reached 90% (range from 78–96%) with five modules, and the addition of a sixth module only increased VAF by 4±0.6% (average over all subjects from UPT and PTB). The dimensionality from the concatenation of all trials for each subject also indicated that five modules are sufficient to reconstruct cutting at reconstruction quality above 80% (86±3%, averaged from all PTB and UPT).

### Motor modules that describe unperturbed and perturbed cutting

**Figure 6:**
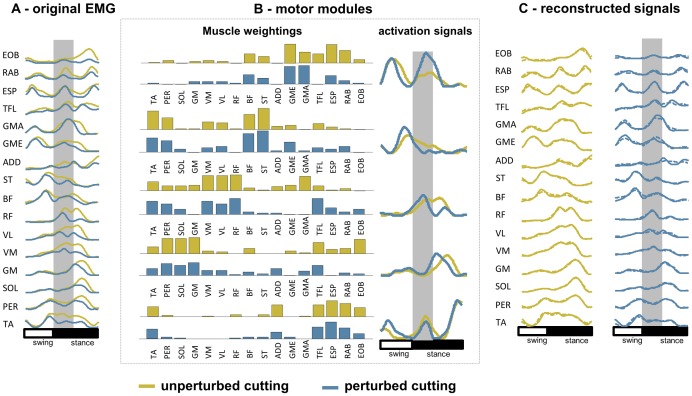
Representative modular organization for perturbed and unperturbed cutting manoeuvres. A) EMG envelopes for unperturbed (*yellow*) and perturbed cutting manoeuvres (*blue*) throughout the cutting cycle. B) muscle activity was processed by a NMF algorithm, which reconstruct the original EMG using a small set of motor modules for both conditions in a similar way. C) original (*solid lines*) and reconstructed EMG from the multiplication of muscle weightings and activation signals (*dashed lines on top of solid lines*).

Aside from the observation that the number of modules was similar for PTB and UPT, the five extracted motor modules for PTB were also similar to those from UPT. By comparing motor modules from UPT and PTB (Figure 6) it can be seen that EMG signals are influenced by perturbations (*panel A*). For this specific subject, it was observed that the muscle weighting for perturbed M1 is slightly different in relation to unperturbed M1 (upper left couple of weightings). The other muscle weightings were not influenced by perturbations. On the other hand, activation signals showed more influences of perturbations during the stance period for all motor modules (*panel B*). Both PTB and UPT EMG were successfully reconstructed by these five motor modules (*panel C*).

### Muscle weightings – inter-subject similarity and effects of perturbations

Similarity indices computed for the muscle weightings among subjects showed median values from 0.77–0.81 (Table 2) with higher similarities found for M3 and M4. In addition, all muscle weightings from UPT were similar to the homologous weightings from PTB (median similarity >0.85, Table 2),

**Table 2:** Median, 25% percentile and 75% percentile of similarities between muscle weightings of different subjects during perturbed cutting manoeuvres (*left*) and similarities between muscle weightings from unperturbed (UPT) and perturbed cuttings manoeuvres (PTB) (*right*) of the five extracted motor modules from all subjects.

	Inter-subject			UPT x PTB		
	Median	25% perc	75% perc	Median	25% perc	75% perc
**M1**	0.77	0.56	0.90	0.87	0.82	0.93
**M2**	0.76	0.57	0.90	0.86	0.78	0.91
**M3**	0.79	0.60	0.92	0.88	0.79	0.92
**M4**	0.77	0.58	0.91	0.88	0.79	0.94
**M5**	0.81	0.62	0.93	0.87	0.69	0.93

### Activation signals – inter-subject similarity and effects of perturbations

Inter-subject similarity computed for activation signals showed median values from 0.51–0.57 (Table 3). In addition, similarities between activation signals from UPT and PTB were found only for M3 and M4, whereas the other motor modules did not show similarities when the whole sample was taken into account. On the other hand, the 75% percentiles for the activation signals in Table 3 demonstrate that some subjects may present high similarity between UPT and PTB.

**Table 3:** Median, 25% percentile and 75% percentile of similarities between activation signals of different subjects during perturbed cutting manoeuvres (*left*) and similarities between activation signals from unperturbed (UPT) and perturbed cuttings manoeuvres (PTB) (*right*) of the five extracted motor modules from all subjects.

	Inter-subject			UPT x PTB		
	Median	25% perc	75% perc	Median	25% perc	75% perc
**M1**	0.55	0.40	0.71	0.73	0.59	0.83
**M2**	0.57	0.42	0.74	0.69	0.53	0.81
**M3**	0.51	0.45	0.70	0.80	0.62	0.88
**M4**	0.55	0.49	0.68	0.87	0.75	0.93
**M5**	0.52	0.48	0.67	0.77	0.60	0.86

### Concatenated motor modules to explain strategies to postural reactions

**Figure 7:**
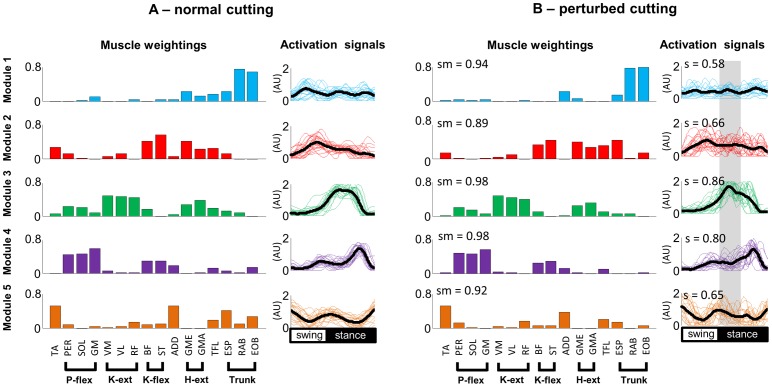
Motor modules that describe cutting manoeuvres without perturbations (A) and with perturbation (B). Abbreviations of the muscle nomenclature are described in the Methods (Section 2.3). The ‘sm’ is the similarity computed between the motor modules from unperturbed and perturbed cuttings. The ‘s’ is the similarity computed between the activation signals from unperturbed and perturbed cuttings.P-flex: plantar flexors; K-ext: knee extensors; K-flex: knee flexors; H-ext: Hip extensors.

Figure 7 shows the concatenation of all subjects for UPT (Figure 7A) and PTB (Figure 6B). In line with the averaged motor modules from Figure 5, the concatenation also shows similar weighting coefficients when comparing UPT and PTB. The lowest similarity among weighting coefficients was found for M2 (0.89) for which hip extensors, ESP and EOB were also activated in this module in response to the perturbation event. The activation signals showed similarity only for M3 and M4, whereas the other three modules were influenced by the perturbation event.

### Reconstruction of perturbations from the unperturbed motor modules

**Figure 8:**
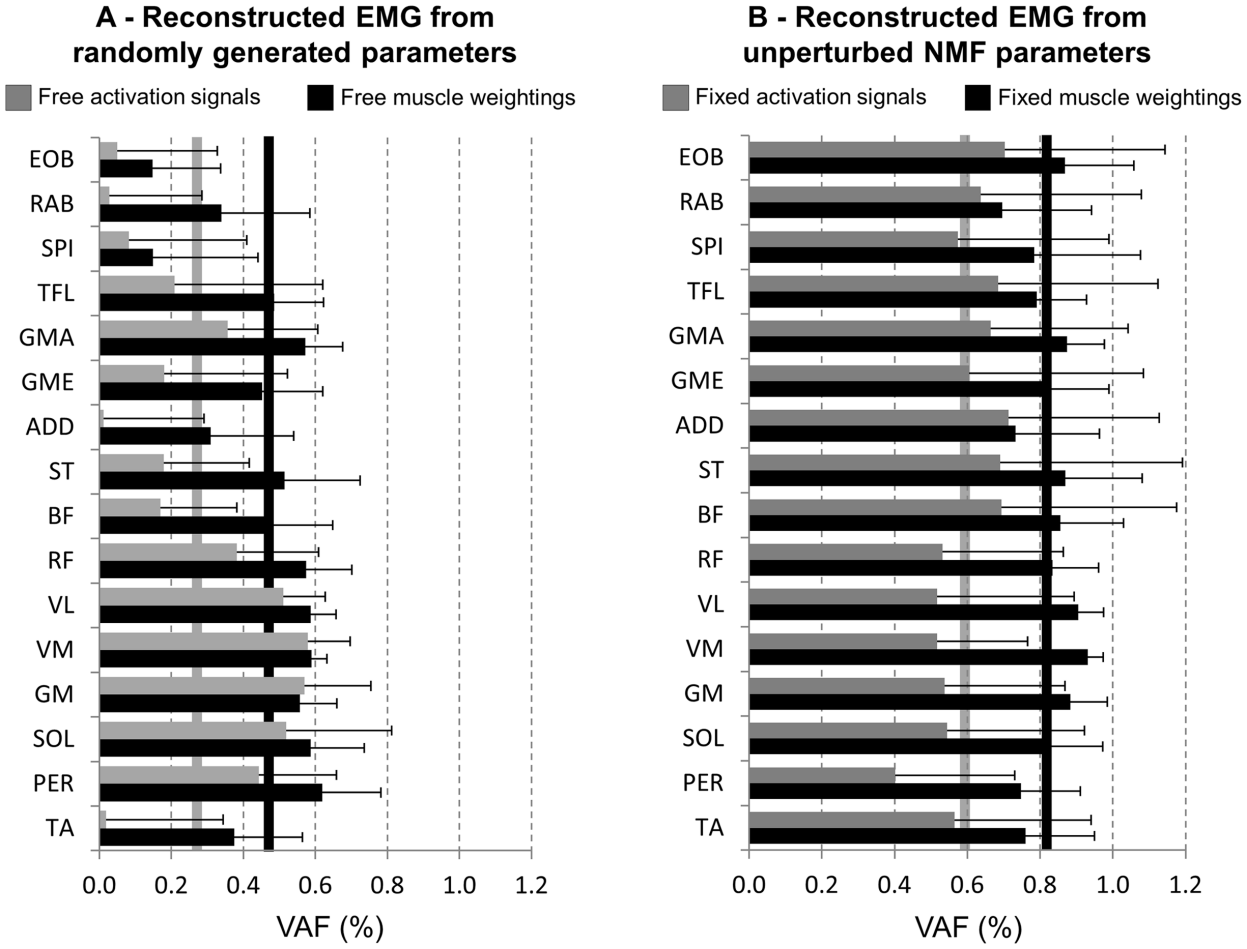
Mean (SD) variance accounted for (VAF) based on the reconstruction of EMG signals from the perturbed cutting manoeuvres in two different methods. IN panel A, VAF between the original perturbed EMG signal and reconstructed perturbed EMG generated from unperturbed muscle weightings and activation signals free to vary (*gray bars*), as well as reconstructed perturbed EMG generated from unperturbed activation signals and muscle weightings free to vary (*black bars*). In Panel B, VAF between the original perturbed EMG signal and reconstructed perturbed EMG generated from perturbed muscle weightings and fixed unperturbed activation signals (*gray bars*), as well as reconstructed perturbed EMG generated from perturbed activation signals and fixed unperturbed muscle weightings (*black bars*). In both panels, vertical bars indicate averages across all muscles for the respective VAF indicated by the color.

Original surface EMG signals from the perturbed conditions were compared to reconstructed EMG data, which were generated in different ways. Initially, unperturbed muscle weightings were multiplied by five activation signals free to vary (EMG_AC_). Another simulation involved unperturbed activation signals and muscle weightings free to vary (EMG_MW_). By using activation signals free to vary in EMG_AC_ the VAF when compared to the original perturbed EMG was 25±44% (Figure 8A, *gray bars*). In addition, when the muscle weightings were free to vary in EMG_MW_ the VAF when compared to the original perturbed EMG was 46±39% (Figure 8B, *black bars*). The EMG reconstruction from free NMF parameters was lower for muscles from the trunk, in comparison to muscles from the lower limb.

The perturbed surface EMG was reconstructed in two other different ways: fixing perturbed muscle weightings and using unperturbed activation signals (Figure 8B, *gray bars*), and fixing perturbed activation signals and using unperturbed muscle weightings (Figure 8, *black bars)*. It was observed that reconstruction of perturbed EMG from unperturbed activation signals resulted in VAF = 59±11%. On the other hand, the reconstruction from unperturbed muscle weightings resulted in a higher VAF (0.82±6%). In both cases, VAF showed consistent values among muscles.

### Perturbation effects on the activation signals

The peak timing of the activation signals related to the stance phase of cutting were not different when comparing UPT and PTB (p>0.05, Table 4). In addition, the time duration between the peak timing from M2 to M3 and from M3 to M4 of UPT cutting task were also not statistically different when compared to PTB (p>0.05).

**Table 4:** Peak timing for the activation signals (% of cutting cycle) of the three motor modules related to the stance phase of unperturbed (UNP) and perturbed (PERT) cutting manoeuvres.

	UNP	PERT
**M2 (% cycle)**	37.2±6	38.5±14
**M3 (% cycle)**	64.1±9	65.7±10
**M4 (% cycle)**	82.9±4	81.6±9
**M2-M3 (% cycle)**	27.6±8	27.2±14
**M3-M4 (% cycle)**	16.7±10	17.0±12

M2-M3: time period from the peak activation of M2 to the peak activation of M3; M3-M4: time period from the peak activation of M3 to the peak activation of M4.

**Figure 9:**
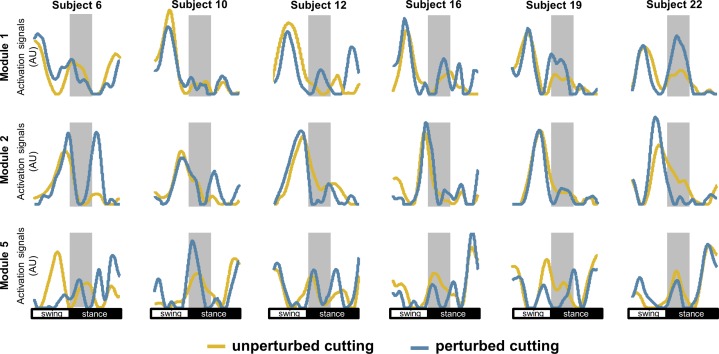
Activation signals extracted from non-negative matrix factorization for unperturbed cutting manoeuvres (*yellow*) and perturbed cutting manoeuvres (*blue*). The illustrated activation signals are respective to module 1, module 2 and module 5 from six subjects of the sample, showing some slight changes caused by perturbations during the task.

Selected activation signals for M1, M2 and M5 from UPT and PTB were compared for six individuals (Figure 9). The predominance of changes in the timing occurred during/after the perturbation period, with minor changes during swing. The M1 showed a second peak activation during the perturbation period when perturbations were elicited. In addition, M2 (related to initial contact modulation) showed reduced activation from initial contact (*the grey in the figure area limits the perturbed period*), which might reduce the activation of hamstring muscles in the early period of stance. M5 showed no constant pattern between subjects, for both PTB and UPT.

## Discussion

The main findings of this study were that small perturbations to balance delivered during fast changes in direction influence the modular control of the task. We observed slight changes in the muscle weightings, and more relevant influences of perturbations were found in the timing properties of the modular control (i.e., the activation signals). Furthermore, the use of fixed muscle weightings to reconstruct perturbed EMG revealed higher and acceptable reconstruction quality in comparison to fixed activation signals, reinforcing the more relevant influence of perturbations on the activation signals. Most likely, the potentiated afferent inputs during perturbations may instantaneously reduce muscular activation during load acceptance for specific motor modules. These results suggest that perturbations to balance during changes in direction reduce the recruitment of muscles related to knee/hip stability at the perturbation event, and that the CNS might not be able to counteract instantaneously to threats occurring at the periphery by means of muscular recruitment in non-trained subjects.

The neural control of locomotion tasks such as walking and running has been described by a modular organization, in which a low-dimensional set of motor modules account for the activation of the main lower limb/trunk muscles [18], [41]. However, only a few investigations were conducted concerning changes in CNS strategies to control locomotion in PTB [1], [20], [42], [43]. Afferent input to the modulation of gait is considered essential, and its role is even more remarkable when balance is challenged. Previous studies have reported increased afferent responsiveness and consequent altered muscular activation during locomotion over slippery surfaces [1] or perturbations such as stumbling [42] and absent support surface [44]. In addition, afferent contributions during walking have also been recently suggested as the main cause for the changes in lower limb activity throughout translational perturbations such as slips [20]. The present results are in line with these previous reports, since the most relevant changes in the modular control were verified in the activation timing of the motor modules. Strong afferent inputs from the perturbation event were integrated with supraspinal descending commands in order to control lower limb joint at the perturbation in the most appropriate way. These commands are subsequently directed to less influenced muscle weightings involved in specific biomechanical goals related to changing direction while running. Robust muscle weightings and slightly varied activation signals according to the task demands have been described in previous perturbation studies, such as during perturbations while standing [45]. Therefore, the current results reinforce the concept that unexpected perturbations may mainly introduce changes in activation signals. Moreover, the absence of changes in the peak timing for the activation of motor modules may further suggest that the central nervous system prioritizes control of functional goals during locomotion in a time-invariant manner, as previously suggested for the addition of voluntary movements during walking [46], as well as for human walking and running at different speeds [27].

Although similar motor modules were found between conditions, the reconstruction accuracy of motor modules from PTB based on fixed activation signals from the UPT does not allow for acceptable reconstruction quality, however reconstruction based on muscle weightings was successful (Figure 8B). Thus, this cross-validation procedure may suggest that the activation signals are indeed the relevant parameters altered by perturbations. Moreover, these outcomes from the cross-validation provide information that is not achievable by the use of random data, as demonstrated by extracting the VAF from the original EMG in comparison to reconstructed EMG in which both activation signals and muscle weightings were free to vary (VAF below 50%). A robust modular organization to perform cutting manoeuvres across subjects has recently been described [21], in which the median inter-subject similarity for the muscle weightings was 0.86. This inter-subject similarity is comparable to those reported for other locomotor tasks such as cycling (similarity ∼0.90, [47]). In the present investigation, the same analysis applied to PTB cutting manoeuvres revealed a slightly reduced similarity (median  = 0.75). This may reflect some differences in the neural strategies to avoid falls, such as previous experiences unexpected perturbations as well as slippery surfaces [48], [49]. Activation signals were influenced to a greater extend, and consequently the similarity between UPT and PTB was reduced (Table 3). However the inter-individual variability has to be considered, since some subjects showed reasonable similarity (e.g., 75% percentiles above 0.8), but in general subjects did not show acceptable similarity.

Activation signals related to M3 and M4 were preserved, which modulate limb extension and forward propulsion by the calf muscles, suggesting that the motor patterns that drive these biomechanical goals during fast changes in direction are robust and may resist perturbation events. The changes in the activation signals for M2 (hamstrings/gluteus activity) reduced muscle recruitment while the perturbation was occurring. These effects were additionally found in traditional EMG analysis in fixed time-window for individual muscles (Figure 2) and eventually reflected in reduced CCI and CCR calculated shortly after initial contact during PTB trials. On the other hand, there was increased excitation in M5 (TA, ADD, TFL, ESP) immediately following the perturbation (Figure 8). For both cases, these changes may be linked to the fact that the supporting limb is unloaded, which leads to a reduced co-contraction of trunk and lower limb muscles [50]. Subsequently, a greater co-activation occurred, possibly attributable to monosynaptic stretch reflexes after unexpected perturbations [3], [22], [23], [51], which may have induced/increased the stiffness in the hip and knee joints, allowing a safe completion of the movement [10], [44], [48]. However it is difficult to clearly separate reflex components from the voluntary actions in factorization analysis such as NMF [20], and assumptions concerning the specific participation of reflex components must be carefully interpreted. In addition, the calculated CCI and CCR during cutting manoeuvres involved EMG signals from BF muscles, which is biarticular and is concomitantly acting at the knee and hip joints. Although previous investigation have found that hamstrings muscles may act at the knee joint as stabilizers independently of their role at the hip [52], our findings must be carefully interpreted.

The modularity found in the present investigation is in agreement with previous reports on running [41] and cutting manoeuvres [21], [53], in which five modules were sufficient to describe the neural control of fast changes in direction during running and a more detailed discussion concerning the neurophysiological meaning of the motor modules has been provided in the respective papers. The perturbation event in the present investigation, however, did not change the modularity of the task, suggesting that the CNS can solve the unpredictable event without increasing the complexity of the control strategy. This result corroborates those reported in our recent investigation [20], in which the number of modules remained unchanged during perturbations, but one module was reorganized for perturbations forward, leftward and rightward in order to regain balance and continue walking. In addition to the changes in one motor module, the activation signals were substantially altered for most motor modules, most likely caused by afferent input received throughout the perturbation event.

Perturbations to balance in the present investigation did not cause substantial changes in hip and knee kinematics. Even though, a reduction in hamstrings EMG activity while sliding, as well as increased knee extension and abduction moments were found. These biomechanical alterations have been previously related to knee injuries in recreational sports practitioners and athletes, and verified in different experimental protocols [54]–[56]. It is believed that reduced hamstrings activation during knee extension may expose the ligamentous structures to higher anterior shear forces, increasing risk of sustaining injuries such as ACL ruptures [6], [12]. Cutting manoeuvres require a high level of stability in the knee joint that might be compromised by reductions in muscle activations during a perturbation event (Figure 3). Despite the fact that perturbations reduced joint moments during load acceptance, the joint moments in the frontal plane were increased for the hip and knee (adduction moment for both joints), concomitant to increased CCI and CCR. These results suggest that small slips while cutting can change the overall joint mechanics and influence the neural control of the lower limb muscles just after the perturbation [50].

Methodological limitations in eliciting perturbations during high speed movements with a change in direction might be the reason to the lack of investigations in this topic. Being aware about these limitations and risks, we elicited harmless 10 cm translations to assure safety with no falls and/or related injuries being reported during the whole experiment. Slips while performing running or cutting manoeuvres might easily overcome 10 cm, requiring stronger postural reactions that may differ from the described reactions in the present results. Such a small translation must be considered as a methodological limitation in order to assure safety. In this way, computer-based simulations may be the best approach in order to understand the possible underlying mechanisms related to postural reactions in such delicate conditions. Even though, our results reinforce the knowledge on the importance of neural commands to the muscles during hazardous events by suggesting that slips strongly influence the neural control of dynamic tasks.

In summary, small perturbations to changes in direction while running elicited mild biomechanical changes during the stance phase. Even though, perturbations influenced the modular organization to control the task, with minor effects on muscle weightings and more prominent changes in the activation signals. The timing properties that control motor modules were likely influenced by the integration of descending commands and afferent inputs from the perturbations. Moreover, reductions in co-contraction ratio for the knee joint muscles, and increased knee abduction moments suggest reduced protection from the neural mechanisms and consequently that the risk for injury might be increased in more severe perturbations.
